# Book Reviews

**Published:** 1805-07-01

**Authors:** 


### CRITICAL ANALYSIS

OF THE

### RECENT PUBLICATIONS

ON THE

### DIFFERENT BRANCHES OF PHYSIC, SURGERY, AND MEDICAL PHILOSOPHY.

"*Coup d'Œil sur les Revolutions et sur la Reforme de la Medicine; par* P. J. G. CABANIS, Membre du Sénat Conservateur, &c. Octavo, pp. 438. Paris, An. xii.—1804."

WE feel considerable satisfaction in laying before our readers this work of M. Cabanis, which, although published only lately, was composed about ten years ago, at the suggestion of Garat; who was, at that time, Commissioner of Public Instruction, and who was busily employed in organizing, on an enlarged and comprehensive plan, all the branches of education. It was, however, the original design of the author, after having given a rapid sketch of the different revolutions in medical science, and pointed out, in a summary manner, the general principles which should guide and direct its reform, to have considered very fully the application of the *analytical method* to the study of Medicine, on which he conceives its farther advancement, in a great measure, to depend. The desire of accomplishing this object retarded the publication of his work, till loss of health deprived him of both the hopes and means of completing his design, and induced him to lay before the public the introduction of his work, the only part which he has been able to finish. The performance, however, even in its present state, must be regarded as a very valuable acquisition to medical literature; and although it may not recommend itself by the novelty of the subject, yet it distinguishes itself, in an eminent degree, by the justness, originality, and discrimination of the views it contains, and bears decisive marks of a bold and penetrating genius. We regret that our limits prevent us from entering into a detailed analysis of the work and confine us to a few extracts, which, however, it is hoped, will serve to justify our opinion of its merits.

"At a time," (says M. Cabanis, after some preliminary remarks on the necessity of a medical reform) "when all the branches of science are, in some measure renewed, those physicians who are endowed with any portion of philosophy, should regard it as a duty to unite their efforts for accomplishing this great reform of their science and their art. The enlightened state of the age will allow it to be more complete and more permanent in its effects, than even Hippocrates could have rendered it in his time. In that rapid and progressive impulse which has been communicated to all the the branches of human knowledge, it would not be sufficient to execute the reforms, which are required merely for the present moment; we should endeavour to pave the way for those which, in the course of time, may become necessary; for all ought to be directed by the same spirit, if not executed on the same plan. Witnesses of the daily advances which are made in the other branches of physics, to which men of genius have applied the true methods of inquiry, physicians surely cannot be excusable, if they suffer the beautiful and extensive science which they cultivate, to remain buried under that crude and undigested mass of materials, which observers have so often collected without discrimination, and which theorists have applied to practice without judgement or examination. Surrounded, as they are, by objects so various, so fugitive, so delicate, and in the examination of which the least aberrations of reasoning or deduction lead to the most dangerous errors, they certainly will not be permitted to tolerate a vague and inaccurate language, capable of obscuring the most simple truths, and of giving to the mere fictions of the imagination all the appearances of reality. The time is come when Medicine shall be placed on a footing with the other sciences, and when their mutual relations shall be determined with precision. Placed between Physics and Moral Philosophy, it is of peculiar importance to ascertain and point out distinctly its true relations to each of these sciences. It must borrow the strict and precise language of the former, and the liberal and communicative spirit of the latter. It must take advantage of all that the intellectual philosophy has most rigorously established in its theories, and of all the nice and delicate illustrations which its application to the sensitive frame presents to view. In fine, after having, by the sure methods of observation, experiment and induction, reduced its principles to a regular system, it will be necessary that the improvements in the plan of instruction, should form and fit for practice a set of minds, at once profound and comprehensive, firm and supple, who join to the light of a superior understanding, that knowledge of life and manners, and that facility of action, without which all the gifts of nature and of art are almost entirely useless. Happy combination, perhaps even indispensable, for preventing the practice of a science, of which the subjects are so various and so delicate, from becoming a mere scourge of humanity." P. 5—7.

The following observations on the discovery of the circulation of the blood, appear to us worthy of notice:

" The new light which was thrown upon the animal economy by this important discovery, served only, we may affirm, to redouble the rage of systems. Nothing else was thought of, but to cause the blood to circulate more freely, to destroy its viscosity, to draw off from the body that which was supposed to be corrupted, to purify it, correct it, and renew it, and to preserve the blood-vessels in a relaxed and pervious state. Hence those torrents of aqueous and diluent drinks, with which Bontokœ and his adherents inundated dated their patients. Hence that sanguinary fury, which the partisans of Botalli thought themselves entitled to put in execution, in their treatment of all sorts of diseases; a fury, which although so often damped by systematic murders, has only ceased for intervals, and still from time to time reappears in the schools. Hence, too, that wretched mania of the transfusion of blood, of which the practice almost always deprived those, who had the temerity to subject themselves to so dangerous an operation, of their reason, or their life.

"Thus, one of the most beautiful discoveries of modern medicine, far from elucidating the practice of the healing art, as there was every reason to expect, only had the effect of misleading weak imaginations, dazzled by its splendor. And it may even still be doubted, whether its application to the knowledge and cure of internal diseases has been of any real use. In surgical cases, even where its assistance is most generally regarded as indispensable, might not observation almost always supply its place? And must we not limit its importance to the elucidation of a point in Anatomy and Physiology, very curious, no doubt, in itself, but which, if it did not indirectly affect many other interesting questions regarding the animal economy, would, perhaps, have contributed very little to our knowledge of its true laws.

"However, under this point of view alone, the discovery of the circulation has rendered services, by which the practice of Medicine has eventually profited, and the glory of its authors can no longer be contested, by the most ridiculous envy, or the most absurd and inconsiderate attachment to paradox." P. 166—168.

We shall subjoin the following remarks on the improvements of Medical education.

"It must be sufficiently evident, that the views, which direct the reform of Medicine, are the same with those which should govern and regulate its instruction. They alone can supply a good plan of education, and a good system of lectures in every branch of it. One very essential point to be attended to, is always to present, to the pupil, the objects in their most natural order, i. e. to begin with the first or most easily known, and, by their assistance, slowly and gradually to proceed to those, which require more profound attention, more skillful examination, or, perhaps, even new methods of research. It ought to be the study of the teacher to develope the ideas of his scholar in the order of their formation, or in the same order in which the objects conjunctly, and their parts in detail, are presented to our view. And the pupil, in particular, after having seized the chain which unites them, must review it from the first to the last link, taking care not to overlook any of the intermediate links, which the mind does not immediately, and, as it were, necessarily, suggest." P. 307—8.

"It is in the eighteenth century, that the system of instruction has made real progress. To the jargon of the schools has succeeded a language, more pure and more precise. The improvement of of mathematical methods; the employment of more accurate means of investigation in physics and in natural history; that philosophical spirit, which, by degrees, has become universal; that refinement and taste, which the numerous master-pieces in literature and the arts have rendered a sort of want, for the polished class of society in every country, have at last, forced the schools to cast off their barbarous rubbish. Reason surrounded them, and assailed them on all sides; and even took possession of their benches. Justice is now done them. They have long bravely combated against common sense, and we may even perceive, that their impotent remains would be ready to renew the contest; but reason has conquered; folly has been overthrown, and, in spite of all its efforts to rise again, must for ever remain so. The duration and the obstinacy of this shameful conflict are precisely the circumstances which preclude the possibility of a return to ancient methods or to ancient errors, in favour of which latter alone the former are of value in the eyes of a certain description of people. Doubtless those men, who are the firm and faithful votaries of truth, will, in all ages, be attacked by ignorance, and persecuted by hypocrisy; but the triumph of their cause is for ever secured. Many branches of human knowledge have attained a sort of perfection; a rich store of materials is collected for others; it only remains for us to apply the true methods of research to all equally; and in particular, to apply them with equal strictness to all the branches of instruction." P. 182—3.

The section on the alliance of Moral Philosophy with Medicine, contains so much just and forcible reasoning, that we are induced to transcribe it at full length.

" We have already shewn," says M. Cabanis, "that all the intellectual sciences should be founded on the physical knowledge of the human body; but we should have a very imperfect idea of the latter, if we neglected to study those organic functions which conspire to produce thought and volition, and to take into view that influence which both these operations exert on the whole, or on particular parts of the sensitive frame. Thus we see, that metaphysics and moral philosophy are equally necessary for the physician. On the first of these we have already sufficiently expatiated. With respect to the second, as it is almost constantly connected with the details of practical medicine, it seems as if it were the sister, rather than the attendant, of the latter. The errors of the imagination, or those of the passions and desires, are evidently the causes of a great proportion of the miseries of man. The diseases even to which he is subjected, generally depend on his own errors, or on those of society, and are always liable to aggravation from the depraved state of his moral constitution. How much may erroneous opinions and irregular desires disturb the functions of the system! How many vicious habits may they not generate in the different organs of the body! and if it be true, that vice, like insanity, is often but a physical disease, how often, in their turn, are diseases diseases produced, either by insanity, which, generally speaking, may occasion disorder in all the living actions of the system; or by vice, which in reality is merely a variety of the former.

"The physician is doubtless unfit for his profession, who has not learned to read in the human heart, as well as to recognize the presence of the febrile state; or, who in treating a diseased body, cannot distinguish, in the features, in the looks, or in the speech, of his patient, the signs of a disordered mind, or of a wounded heart. How can he seize the true character of these diseases, which are concealed under the semblance of mental emotion, or of those several disorders, which exhibit all the symptoms of certain physical diseases? How can he restore its wonted serenity to that agitated mind, to that soul consumed by a gnawing melancholy, if he is ignorant of the organic lesions, which these moral affections may produce; and of the derangement of the functions with which they are generally connected? How can he revive the dying flame of life, in a body drooping, or devoured by anguish, if he knows not what sufferings he must first alleviate, and what idle dreams he must first dispel?

"Doubtless, it is the duty of the physician to afford the sweetest and most soothing consolations to the patient couched on the bed of sickness; it is he alone, who can penetrate farthest into the confidence of infirmity and misfortune, and therefore it is he who can pour the most salutary balm into their wounds. But, for the same reasons, he must not remain ignorant of the nature and destiny of these unhappy and too feeble mortals; he must not be void of compassion for those errors and miseries which so readily may become the lot of every one; but he must be indulgent and kind, as well as circumspect and reasonable. Every one else may hate vice, and be revolted at folly; but the physician, if he knows how to observe and judge properly; if he possesses good sense, if he is just and liberal in his sentiments, can only feel pity for both; and can only redouble his zeal for the service of those degraded and unfortunate creatures, who ought to excite his compassion more forcibly, the more that they are insensible to their own unhappy state.

"Who has not had an opportunity of seeing some unfortunate beings, victims of their own fatal passions, drag on a languishing existence towards the tomb, while they sought for some expressions of concern, rather than for life? Who has not observed the cruel agitations of those terrified imaginations, who tormenting themselves with the tortures of their own fancy, combine sometimes, with this mania, sentiments of the sublimest virtue? Can there be a more grateful enjoyment, then, than to allay those groundless alarms, those fictitious terrors, to cause the voice of reason to be heard amidst their numerous perplexities? Those persons who evince the greatest sensibility and compassion towards others (and such persons are most liable to all sorts of error) surely deserve the most particular attention on the part of the virtuous and feeling physician.

cian. Could any one, who is not completely ignorant of the sentiments which form the human heart, could any one avoid being deeply affected at the sufferings of those, who never witnessed the sufferings of others, without feeling an anxious wish to relieve them? Could they be sparing of their care and attention to those who live only by their social affections?

"But to return to considerations more strictly medical, we may observe, that methods of cure, which often are simple and uniform, when applied to individuals, whose minds, or whose sensibility, have received but little culture, become very complicated, various, and difficult, when directed to persons whose faculties have been more completely developed. How many fibres may be agitated by the slightest causes, when the mind receives and combines a variety of impressions, and when many vivid emotions animate the breast! Not to speak of the habits which are contracted by application to different trades; whenever one emerges from the sphere of mere animal life, whenever one becomes separated from the common class of society, the treatment of each disorder demands particular combinations of ideas, and frequently combinations of ideas which do not relate to the disease itself. Thus, the practice of Medicine is reduced to a few simple rules in the country, and in hospitals; but it is obliged to multiply, to vary, and to combine its resources, when applied to men of business, to men of letters, and to artists, and to all persons whose lives are not devoted to mere manual labour." P. 420—5.

---

*The Edinburgh Medical and Surgical Journal, exhibiting a concise View of the latest Discoveries in Medicine, Surgery, Pharmacy, &c. No. 2. April,* 1805.

At length the 2d number of this useful publication has appeared; and if the table of contents is less showy, it is at least adorned with as respectable names: if the original communications occupy less space, they are more numerous; and their want of bulk is supplied by an anonymous communication. We trust, the encouragement of the public will keep pace with the industry of the managers; and also, that such encouragement will increase, instead of diminishing their exertions.

The first paper may be called a continuation of the fifth of the last number, being a communication from Mr. Astley Cooper, of the deficiency, in a female, of all the parts anterior to the urinary bladder, and of the anterior portion of that viscus, the posterior part projecting through the aperture in the abdominal muscles, where it formed a tumour, on the surface of which the ureters opened. As this case can afford no practical remarks to the faculty in particular, we shall transcribe the introductory part, as a moral lecture to every individual in this christian country.

"Ann

"Ann Carter, aged 22, was admitted, on the 4th of March, 1803, into St. Thomas's Hospital, under the care of Mr. Cline, on account of a fungous tumour on the lower part of the abdomen, through which the urine was involuntarily discharged. This misfortune she had laboured under from her birth; but when she arrived at the age of puberty, her situation, in that part of the country in which she was born, became insupportable to her feelings, on account of her infirmity being known. She therefore resolved to seek her subsistence in London, where she vainly imagined it might escape detection, and she soon procured the situation of a servant to a family in town; but being unable to retain her urine, her clothes were rendered offensive by it, and her bed was nightly wetted. Her infirmity was thus soon discovered by her employers, and she was dismissed from their service. By frequent disappointments of this kind, she was reduced to seek a miserable subsistence by begging, and spent days and nights in the streets of London, until, becoming ill, from the effects of want and exposure to the severity of the weather, she was carried, by the police, to an hospital; and when admitted into St. Thomas's, she was so much reduced, by poverty and sickness, that she died on the 8th of March, four days after her admission."

An account of the dissection follows, with the accuracy we might expect from such a source, illustrated by a wooden sketch, which we think, even in such materials, need not have been so coarse and confused.

This history is followed by a conclusion of the paper before referred to, and contained in the 1st number. Though there is nothing objectionable in the remarks, yet, we are of opinion, many of them might have been spared. The principal merit consists in the diligence with which so many cases have been collected from various sources, by which the reader could not fail to mark the general analogy, without the assistance of the compiler. We hope we shall be excused giving a gentle hint on the application of terms. When we read, "Mr. Cooper mentions, if Ann Carter got any thing to drink," we could not help referring to the passage, to see if such were his words. We mention this, because, though no one who is acquainted with the English language and Mr. Cooper, will suspect him of using such an expression, yet with others it may pass for an authority.

The third article is entitled, "Observations on the Yellow Fever of America, tending to prove, that it does not depend on any peculiar modification of the atmosphere; that it is not preceded by any malignant change in the type of other diseases; and that it is not attended with carbuncles, or glandular swellings. By Dr. James S. Stringham, Professor of Chemistry in Columbia College, New York."

This paper, at least, shows the great difficulty of arriving at truth, if no other advantage can be drawn from it. It is strange that there should still be any kind of doubt, relative to the exact description description of a disease which so regularly occurs, and which so many physicians have witnessed in every stage. We shall not, however add to the embarassments which may arise from these differences of opinion; for if men on the spot cannot agree on subjects the most obvious to the senses, it is not likely that we, at such a distance, should reconcile them: we only wish, that they would be careful to take notes whilst engaged in the treatment of their disputed cases; or, if they have taken notes, that they would favour the public with them.

"Observations on the Muriate of Lime, in the Cure of Scrofula, and other States of Debility. By JAMES WOOD, Physician to the Newcastle Infirmary and Dispensary."

DR. WOOD scruples not to assert, that, in many respects, muriate of lime possesses advantages over muriate of barytes, in the same proportion that the vaccine does over the variolous inoculation. Besides its much more certain efficacy, the facility of exhibiting it is equally great, since we may give a full dose of the muriate of lime, at first, without the cautious gradation which so much retards the benefit we expect from the former composition. Some directions follow, relative to the manner of preparing and exhibiting the remedy.

"Case of an encysted Tumour occupying the greater Part of the posterior Portion of the right Hæmisphere of the Brain. By P. BATEMAN, M. D. F. L. S. Physician to the Carey-street Dispensary, and to the Fever Institution."

THIS is, in many respects, a very valuable case; for though no practical inferences can be immediately drawn from it, yet it adds, at least, some little towards the pathological knowledge of a part the most important, and the most obscure. The encysted tumour is described as filled with pure *pus*. The cyst was firm, and easily removed, extremely sanguiferous, and scarcely attached to the brain. All the diseased appearances were on the right side; but the paralysis, for some time, confined to the left extremities. The following remarks contain all the inferences we can draw; and are so well expressed, that we shall give them in the author's own words.

"The preceding case is not offered with a view of deducing from it any general conclusion. It seemed valuable, on account of the connexion of the symptoms with the morbid state of the organ, as ascertained by examination after death; and, on that account, worthy to be recorded. We are yet very ignorant of any marks of discrimination, in the living body, of pressure or irritation, as applied to that very complex organ, the brain. The relations of the older anatomists have thrown little light upon this subject, in consequence of the imperfect accounts they have left us of the symptoms. There is a case well related by Platerus, and copied by Bonetus in his Sepulchretum (Sect. *De oculi effectibus*, Obs. I.)

which, which, in the course of the symptoms, somewhat resembles the foregoing case. A tumour, of a smaller size, and greater solidity, than the one before us, being of a substance like the coagulated white of an egg, was found seated in the *anterior* portion of the right hemisphere; convulsions, nevertheless, occurred. From six very valuable cases, which are too little known, from their being published in a work of very limited circulation among medical practitioners (Transactions of the Royal Society of Edinburgh, vol. ii. p. 17) Mr. Anderson, surgeon, in Leith, has drawn the following conclusions.

"1. When one hemisphere of the brain is affected, it generally produces morbid symptoms on the other side of the body.

"2. When both hemispheres are affected, the whole body suffers.

"3. Though only one hemisphere is affected, when the injury is great, the whole body suffers.

"4. Though the cerebrum alone is hurt, it produces morbid symptoms in all muscles of voluntary motion, whether their nerves arise immediately from the cerebrum, the cerebellum, or from the medulla oblongata.

"5. In cases of external accident, where one side only is affected, the prognosis is more favourable than when both sides suffer.

"The only other approach to generalization, that I have met with, is, an observation by Dr. Baillie (*Morbid Anat.* p. 457). 'It is worthy of remark here,' he says, 'that, when tumours, of any kind, press upon the thalami nervorum opticorum, or the optic nerves themselves, within the cranium, vision becomes impaired in various ways; and that, when tumours press upon the tuberculum annulare, or the medulla oblongata, convulsions are very apt to occur.' The first part of the observation will, I believe, be allowed by every one to be correct; but the last, I apprehend, is not invariably supported by the records of pathology; and, with the greatest deference to such high authority in morbid anatomy, it may be questioned whether we are yet in possession of a sufficient number of facts to warrant any general conclusion. *Ars longa, judicium difficile.*

Two Cases of Acute Rheumatism, treated with Opium; and some Observations on the comparative Merits of that Method, and of the Treatment which is founded on the Principle of Depletion; communicated by J. J. De Roches, M. D. Physician to the City Dispensary, and Fellow of the Medical Societies of London and Edinburgh.

As each of these patients was purged, and one of them attacked with rheumatism, after chronic diarrhœa, the conclusion of dysentery, we conceive the author's intention could only be to show, that it is possible to evacuate too freely in acute rheumatism, or to mistake the chronic for the acute disease. All this will not, we presume, be disputed. As to the "nosological dislike to opium in pure inflammation," of which the author accuses the Cullenian school, we conceive, some, at least, of these remarks, might have been spared. The principal objections against opium, in inflammatory diseases, is, that it renders the symptoms less to be depended upon. If the inflammation ceases, opiates are unnecessary for relieving pain. If the inflammation continues, they rarely succeed; but interrupt the effect of the other remedies by costiveness. But where high irritability assumes the appearance of inflammation, which is often the case, opium will prove the cure, especially if preceded by common purges. Sydenham's observation is therefore very just, that, in those cases of rheumatism which require frequent bleeding, and such are by no means uncommon, opiates are, at least, of little avail during the paroxysm, which they too often protract. We fear we have given too much room to this communication; because, if the practitioner has not courage enough to be decided, in these cases, no rules can be of much use to him; and because, as the valuable writer, whom Dr. De Roches is disposed to correct, observes, of another disease, there are some cases so mild, that even the blunders of the doctor will scarcely protract the patient's recovery.

History of a Tumour in the Breast, with some Reflexions; communicated by HENRY REEVES, M. D. Fellow of the Medical Societies of London and Edinburgh.

DR. REEVES apologises for the minuteness of his relations. We not only excuse him; but, whilst we are thankful for the particulars he enumerates, we could wish they had been still more. Among other things, we could wish that the appearance of the tumour had been described with more nicety. "It had," we are told, "the appearance of condensed cellular substance, or hardened steatoma." Does this mean, that steatomatous substance was inclosed in separate cells? A few ounces also of gelatinous substance was discharged by puncture. This, we conceive, must have been contained in a cyst, perhaps similar to the cells which contained the steatoma.

Another not unimportant enquiry remains unsatisfied. The patient first perceived the tumour in September, 1803. The following January she married, and, about that time, the tumour began to increase. "She considered herself pregnant too," we are informed; and, that "this change in her constitution might excite the local diseased action, or, at least, accelerate its progress." As she lived till the following September, it would have been easy to ascertain whether she was really with child or not, unless symptoms of abortion should have taken place, which is not mentioned.

Our readers will, perhaps, see our motives for these inquiries. Dr. Adams, of whose treatise, on the cancerous breast, we gave so long and particular an account in one of our former volumes, takes notice of cells filled with a gelatinous substance, and others with steatoma. These he seems to consider as possessing a similar life with carcinoma, and multiplying in the same manner. The case above affords some illustration, at least, of this theory.— Two other remarks are found in the same author. The first, that if the disease attacks a young subject, without a previous injury, it not only proves fatal, as in other cases, but more rapidly so. The second, that in all such cases as have come to his knowledge, the patient has been either single, or without children if married.

Added to this melancholy case, we have that of her sister, who, refusing to submit to an operation which, she saw, had proved unsuccessful, only protracted a more miserable existence.

On the whole, these cases are highly valuable; and if more particulars can be collected, we hope to meet them in a succeeding number.

Case of Cancer of both Testicles, which terminated favourably by the Supervention of Scurvy; communicated by JOHN LIVINGSTONE, Surgeon of the Cirencester.

THIS is a very valuable case, in every point of view. We shall not, therefore, engage in any controversy relative to the nature of the original distemper; but heartily thank the Editors and Mr. Livingstone, for recording the particulars as they are.

Practical Obversations on the Pneumonic Diseases of the Poor; by CHARLES BADHAM, M. D. Physician to the Westminster General Dispensary.

PULMONARY complaints are too much confused by systematic writers. In so uncertain a climate as ours, and in a town where the labouring poor are so much exposed to the vicissitudes of season and weather, they become a most important object of inquiry; and Dr. Badham has shown, that the opportunities the Dispensary has afforded him have not been neglected. His remarks on the frequency of pleuritis in children, and the necessity of copious bleeding, are amply confirmed by occurrences in dissecting rooms; and, we trust, the practitioners of the metropolis are daily becoming more sensible of these truths.

Pleuritis rheumatica next comes under the doctor's notice, and his remarks are not less important; if they are less perspicuous and decided, this arises from the greater intricacy of the subject.

Lastly, those chronic pectoral complaints which, after a few winters, often end in incurable hydrothorax, are well marked. Some general observations follow, on effusions of water into the chest, which the author considers as by no means so generally fatal as Dr. Cullen, and some other writers, have supposed. The frequent fatality of the disease he imputes rather to the previous causes, of which this is only the concluding symptom.

Historical and Critical Analysis of the Functions of the Skin, by Geo. Kelly, M. D. Leith.

This is an useful and ingenious paper; its title speaks its contents. We sincerely hope the author will increase the number of well-authenticated experiments on this important subject.

The last original communication is anonymous; but superscribed, The Enquirer. The first enquiry is, "What are the comparative Advantages of the different Modes proposed for the Treatment of Ulcerated Legs?" The paper contains nothing particular to recommend it.

Such are the original communications of this number. The analytical part follows; on which, we conceive, it becomes us to be silent.

The Medical Intelligence contains an Account of the new Arrangement made in the Medical Department of the Navy;—a Report from one of the Dispensaries of the English Metropolis;—a Recipe for Tinea Capitis;—a long Paper on the Progress of Vaccination;—a Description of a Car for conveying sick and wounded Troops;—a Case of Hernia, in which the gut protruded between the muscles of the abdomen, somewhat above the ring, and afterwards followed the course of the common inguinal hernia, with other peculiarities which the nature of the case produced;—a List of intended Medical Publications closes the number.

---

*An Address to the Medical Practitioners of Ireland, by* Samuel B. Labatt, *M. D. Licenciate of the College of Physicians, and Secretary to the Cow Pock Institution, North Cope Street, Dublin, 8vo. p.* 136. 1805.

This is the best compendium of vaccination we have yet met with. It contains an abstract of all that has been written on the subject, carefully detailing the common appearances, and afterwards marking those peculiarities which sometimes occur, distinguishing such as are no objection to the success of the operation, from such as render it uncertain, or have been found marks of spurious pustules or vesicles.

After an historical account of the discovery, and subsequent practice, of vaccination, the author divides his work in the following manner.

"1. Local appearance and progress of inoculated cow pock, under its most perfect form.

"2. Remarks on its several stages, and the minute differences which are commonly observed therein.

"3. Varieties frequently observed, but which are not incompatible with the genuine disease.

"4. Deviations of greater magnitude, or suspicious cases.

"5. Distinctive marks of spurious cow pock, with suggestions of some of its probable causes.

"6. Constitutional symptoms.

7. Inocula-

"7. Inoculation.—The mode of conducting it, and circumstances to be attended to in the state of the patient, and the medical treatment of the complaint. The best modes of preserving the virus."

Under the last article the author very justly observes, that practitioners have sometimes made too light of the practice; in consequence of which, some patients have been guilty of excesses, and made use of exertions which would scarcely have been prudent, even if virus had not been inserted. We would also add, that, by thus magnifying the simplicity of the practice, and speaking of it as uniformly innocuous, some people have been rendered incredulous as to its prophylactic properties; and others have conceived themselves deceived, or improperly treated, when any unfavourable accidents have occurred.

The work concludes with an apology for the minuteness with which many apparently less important articles are described. For our parts, we have been so little fatigued with perusing this account of a subject so long familiar to us, that we could have been glad if Dr. Labatt had oftener added his own remarks to the authorities he quotes. However, we applaud very much his extreme caution, and shall, on that account, be always particularly happy in receiving his original communications.

---

*An Address, delivered at the Small-Pox and Inoculation Hospitals, on Wednesday, April* 3, 1805, *previous to the Funeral of* WILLIAM WOODVILLE, *M. D. Physician to that Institution, who died there on Tuesday, March* 26, 1805, *by* ANTHONY HIGHMORE, *Secretary. Printed at the request of the House Committee,*

DR. WOODVILLE, it seems, for a few weeks before his death, resided in the Hospital, having no near connections in London to *receive his parting breath.* The present encomium is handsome, without flattery; and minute, without prolixity. It seems to flow from long habits of friendship, originating in continually labouring in the same vineyard, and strengthened by a good opinion, which long acquaintance could not but confirm.

---

### *Dr. Jackson, on the Treatment of Fevers.*

( Continued from vol. xiii. pp. 564. —567. )

"It is a common observation, that excitability is ordinarily connected with, or influenced by, the operation of heat. Hence an increase of the degree of superficial heat, promoted, even by artificial means, presents itself as a cause calculated to ensure effect. The presence of heat, however produced, is also considered as a general index of forming a judgement of the result.

"That the state of excitability, is the cause upon which cold bathing acts as a remedy in the cure of fever, is so clear, as not to require any proof; that heat is frequently an accessory caus*e*

of excitability is also true. The author has always considered the subject on this ground; consequently, where the excitability of the surface was deficient in the actual circumstances of the case, his efforts were uniformly directed to the raising of it artificially to a point, where it becomes capable of an easy impression. It must, however, be remembered, that excitability, or rather susceptibility, is not suppressed or dormant from the operation of one cause only, or expressed in one condition simply: hence the means required to restore it, must have a corresponding variety. Torpor is unfavourable to action of every kind; and torpor, connected with plethora, stagnation in the venous system, and suppression of secretions, is unfavourable to the action of cold bathing. In this case, susceptibility is restored by bleeding. Torpor arises from other causes; and susceptibility is restored by other means, viz. by increasing the heat of the sick apartment, where the weather is cold and damp; by frictions of the skin; and particularly by warm bathing.

"The rule of measuring the heat by a thermometer aims at exactness; but it is defective in application, for it does not touch all the circumstances of the case. It will be found, upon trial, that sensation gives a better idea of morbid heat than a thermometer; in short, measure by sensation is that to which we must at last resort. A thermometer only measures absolute quantity; it gives no information on the subject of quality, whether of the kind consistent with life, or of the kind which indicates the presence of a process leading to disorganization and destruction. It is commonly known, that increase of heat is usually a symptom in fever; and it is admitted, in this place, that a certain condition of increased heat,—but not every condition, furnishes an indication for the employment of cold bathing. It is possible that excess of heat may exist, and it actually does exist, without superficial excitablity; that is, without a due share of sensibility of surface, both in the early period, and in the latter stages of fever. Such condition of fever is common in spring, common with Europeans, soon after their arrival in tropical climates, both at the commencement, and in the after period of the disease, either as connected with plethora, or with internal congestion. The heat is then often ardent,—particularly on the trunk of the body. A thermometer, in this case, proves a fallacious guide. It indicates a high temperature; but experience proves, that cold bathing does no good;—it probably does harm in the case connected with internal congestion. To trials in such cases, it is believed the credit of this remedy has been often sacrificed in the fever of the West Indies, and probably in the fever of America. But further, if an increase of heat, as indicated by the thermometer, or even by sensation, be considered as a circumstance uniformly necessary for the safe and useful employment of cold bathing, the remedy will be denied to that numerous class of fevers, in which the skin is moist and soft, cool, even cooler than natural, though though retaining an equal share of the life and sensibility, which yet continues to animate the frame; a case not uncommon in warm climates, where tremors, startings, and faintings, make prominent features of the disease. Here, washing with cold water, at least with cold salt water, is known to be singularly beneficial. If it then be true, that cold bathing is useful without an apparent excess of heat, and that an excess of heat may exist where cold bathing is not useful, it is evident, that excess of heat, as expressed by a thermometer, even by sensation, cannot be allowed to be the radical condition, on which the benefits of cold bathing in fevers depend; nor the measure of such heat, the rule whereby to sanction its use. Cause and effect are not separable. Cold bathing always produces effect, where there is a quick susceptibility of impression; where that it wanting, it has little perceptible action. But if cold bathing, independently of this, be supposed to cure fever on the principle simply of abstracting excess of heat, the cure of the disease may be conducted in such manner as never to fail; for cold, in its application, is capable of being carried to the point, not only of abstracting excess, but even of extinguishing just proportion. It is, however, fully proved, that morbid excess of heat may be extinguished, while disease remains; or that processes, destructive of life, may go on in fevers, without any unusual extrication of animal heat. Heat is only one condition, or one expression of deranged action; it is not the uniform and positive cause of the disease, which it ought to be, on the supposition, that abstraction of its excess cuts short the course of the diseased motions. This is so evident, that it would not have been necessary to have said so much in explanation of a matter which is clear, did not common opinion rest the value of the remedy on a supposed abstraction of heat. In that view it is only of limited application; it even appears, if there be any faith in experience, to encounter contradiction, in the point of fact, on that ground. On the other, as acting by restoring the natural rhythm of movement in the organic structure, by the force of a new stimulus, it preserves a consistent, intelligible, and clear explanation throughout.

"The management of bathing in the cure of fevers, is a subject so important in itself, that it will be less blameable to repeat instruction, in some parts, than to leave it defective in others. The general condition, with which the effect is so intimately connected, has been noticed, viz. susceptibility of impression in the whole system, and more particularly in the organ, or system of parts, to which the application is made. It now follows, to notice in detail, the forms and conditions of disease, in which the effects of the remedy are beneficial or otherwise.

"As the good effects are merely relative to the condition of the subject, it is evident, that, if the favourable condition does not exist in the actual circumstances of the case, the first object is directed to the means of giving it. In the first form of fever, noticed in this place, viz. that connected with plethora, oppression, tumult, and irritation, (seemingly from resistance in the circulating system;) suspended secretions, a dry, hot, and impervious skin;—a condition frequent in the fevers of spring, even of summer, in very hot and dry weather; and in some manner peculiar to Europeans, newly transported to tropical climates; the affusion of cold water on the naked body, has only a slight and temporay effect. It refreshes for a time; it does not change the nature of the disease, or arrests its course. But while cold bathing, applied uuder the circumstances described, is followed by no material or permanent advantage; yet, where the condition is changed by previous bleeding, carried to the point of arresting the existing movements, and of restoring the susceptibility of impression to other causes; the affusion of cold water, then, gives origin to a train of action, similar to that of health, which, with due care, is eventually confirmed into the healthy habit,—without injury, and without danger of retrograding to its former path. Bleeding and cold bathing, here, furnish a speedy, a safe, and effectual cure for a form of fever, which destroys life occasionally in every country; but which has committed dreadful ravages among Europeans, particularly, among European soldiers in tropical climates. The remedy is comprehended in the means now mentioned; but the effect depends on the management. A scanty bleeding, rarely prepares the condition prescribed for the application of the means; and, unless the condition be duly prepared, the effect is looked for in vain.

"In the second condition of general fever, where there already exists a due share of susceptibility, and more particularly, where there is increased excitability in the surface, the application of cold bathing requires no preparation. The condition exists, and the effect is decisive of health.

"The fevers of the periodic class exhibit great variety of condition; and as the benefits of bathing are relative to condition, so this remedy is sometimes the cause of great good,—sometimes of no value. In intermittents, and remittents not accompanied with signs of internal congestion, in which the hot stage developes freely, tending naturally to a copious and warm perspiration,—cold bathing, properly managed, is capable of eminent good. When the hot stage is completely formed, the application of cold water to the head and shoulders, in repeated affusions, sometimes extinguishes abruptly, generally shortens, the duration of the paroxsym; the succeeding intermission is also more perfect. If care has been taken previously to remove all congestion from internal parts, particularly from the biliary organs, it often seems to suspend the course of the disease, in a similar manner with Peruvian bark. But though this be true, it also ought to be remembered, that where this effect is expected as a result, the expression of the disease ought to be more in the outer circle of the circulation, than in the internal parts; for it is found by experience, that that cold bathing is less useful in the low autumnal bilious fever of European countries, than in most other kinds of acute disease. The expresssion of febrile action is, in this case, little in the channels of general circulation; the excitability of surface is not strong and permanent; while there is often irritation, and sometimes a congestion in the organic structure of the alimentary canal and biliary system. The good effects of cold bathing, in such case, are not remarkable; and the remedy, when employed, requires to be preceded by a particular management, viz.—by warm bathing, by frictions, and warm temperature of air, capable of producing the previous condition, that is, excitability of surface. But even with this management, as the change perhaps can scarcely be rendered complete, the effect is seldom so decisive, as it is in some other forms of malady. Yet, when the condition of a fever so originating, has been changed by accumulation in ships or barracks, or by circumstances of weather and other management, so as to produce a form of disease, the action of which is more particularly manifested in the vascular system and surface of the body, cold bathing resumes its place. Such change often happens, when troops retire from the field into winter quarters; or when they embark in transport ships for foreign service.

"The action of the fever of the contagious character is particularly directed to the skin; consequently the action of bathing, may be supposed to be particularly adapted to its cure. In this form of fever, there are rarely marks of internal congestion:—The first passages—stomach and bowels, receive the first impression of the action of the cause; the superficial parts of the body manifest, more than others, the signs of the first febrile irritation. The skin and superficial parts are differently affected; they are either preternaturally susceptible of impression, with various flying pains and burning sensations; or, they are cold, dull, and torpid. Circumstances so opposite, though fundamentally requiring the same radical remedy, require different means of preparing the previous condition. The first action of the cause of contagious fever, is demonstrably upon the stomach; — a vomit is the first remedy. After the operation of an emetic, the affusion of cold water rarely fails to cut short the course of that form, which is distinguished by heat and superficial excitement. Where the surface is torpid, languid, and greasy; air of a warm temperature, exercise in open air, frictions to the skin, emetics, and warm bathing, as animating and enlivening the surface, precede the use of the final remedy,—the affusion of the cold water. The effect is then, for the most part, complete.

"The good effects of bathing are observed to be most remarkable, where the action of the disease is principally manifested in the surface of the body; they are also considerable, where it affects the muscular parts, or moving powers. Thus, in fevers of the rheumatic form, bleeding, warm and cold bathing, employed in the proper order of succession, and with due attention tention to circumstances, may be almost always considered as means, effective of a safe and speedy cure.

"In fevers, where an internal local affection is a prominent feature of the diseased action, the good effects of cold bathing are very limited;—the application of the remedy is even sometimes dangerous. It has been observed already, that cold bathing has no very decided power over the autumnal fever of European countries. Cold bathing generally applied, has, in like manner, but little effect over the pure dysenteric form; the application of wet cloths, or wet sponges, to the abdomen; and still more, the sitting down in a tub of cold water, or in a running stream, under the torments of this disease, produce singular relief from tenesmus, and contribute materially to the cure of the malady, whether in the chronic or acute stage. It may be concluded from such example, and the truth of the fact is well ascertained, that the benefit of cold bathing actually depends upon direct application to the part preternaturally excited, or to parts connected with it, by direct continuity; for, when the action of the cause of fever is principally manifested in the organic structure of the superficial parts of the body, the change is great, and the good effects are signal. Where this condition is wanting, the same application does no good. On this ground, the application of cold water in glyster, or the application of cold water to the fundament, as a part continuous with the internal surface of the alimentary canal, gives great relief in the dysenteric form of fever; the same application to the body generally, more especially to the extremities, is usually hurtful. The fact is well established, and it is well illustrated, in the example of drenching persons ill of burning fevers with cold drink. It is proved, that in fevers, accompanied with excessive and raging thirst, the coldest water may be drunk with safety, and without limitation of quantity. If the raging thirst be completely satisfied, the fever, or the paroxysm of the fever, will probably be extinguished without danger, in the same manner as a fever, manifesting its action in the surface, is extinguished by cold bathing. On the contrary, when the surface and external parts of the body are preternaturally excited, the limbs at the outmost stretch of their exertions in exercise, or in travelling, the skin scorched, and the body melting under a burning sun, a draught of cold water, drank suddenly at such a time, often occasions death,—instantly, as a stroke of lightning. The excitement is then in the surface; the cold drink is misapplied, for the stomach is faint rather than thirsty.—But if cold drink, in such case, has fatal effects; washing the body with cold water, is safe and refreshing; for the application is direct to the cause.

"From the different views, in which the subject of cold bathing has now been placed, the circumstances, connected with which, have been noticed, and repeatedly verified by the author, the reader may, probably, be enabled to entertain some idea of the mode of operation, and directed into the road of employing it as a remedy upon principle, and with an effect correctly estimated. Its operation consists in exciting a new train of movement, contrary to the existing one, and analogous to that of health. The effect corresponds, in degree of perfection, with the power of the agent, and the susceptibility of the subject; that is, the degree of cold and excitability of the moving principle. It thus often fails, where the power of the agent is weak; as, for example, in hot climates, where the degree of cold cannot be brought near the freezing point; more especially, where there exists, at the same time, a strong morbid impression, blunting sensibility, and rendering the machine less sensible of the impressions of minor causes. The affusion of cold water, judiciously applied to the naked body, at a temperature of thirty-two degrees of Fahrenheit's thermometer, or the rubbing of the naked body with snow, rarely fails to impress the system; to controul the existing movement, though deeply fixed; and to excite a new train of action of a better kind, if the foundations of organization have not been previously loosened or destroyed. The application of snow is powerful; for even where the action of life is suppressed, as in the case of frost-bitten limbs, it speedily restores the part to its natural function. Cold may thus, in certain circumstances, be said to be a power strongly stimulative of animal life. The truth of the fact is familiar.

[ To be concluded in our next. ]

